# Intracranial vasculopathy with MR vessel wall imaging: a case series

**DOI:** 10.1093/bjrcr/uaae036

**Published:** 2024-10-03

**Authors:** Long Hin Sin, Yat Sing Lee, Wai Tat Victor Chan, Chi Wai Siu, Chong Boon Tan

**Affiliations:** Department of Radiology and Nuclear Medicine, Neuroscience Center, Tuen Mun Hospital, Hong Kong; Department of Radiology and Nuclear Medicine, Neuroscience Center, Tuen Mun Hospital, Hong Kong; Department of Radiology and Nuclear Medicine, Neuroscience Center, Tuen Mun Hospital, Hong Kong; Department of Radiology and Nuclear Medicine, Neuroscience Center, Tuen Mun Hospital, Hong Kong; Department of Radiology and Nuclear Medicine, Neuroscience Center, Tuen Mun Hospital, Hong Kong

**Keywords:** magnetic resonance imaging, vessel wall imaging, computer tomography, vasculopathy, vasculitis

## Abstract

Conventional luminal imaging, often the first line investigation, has helped physicians in diagnosing countless patients with cerebrovascular disease but regrettably, it offers little diagnostic clues in patients with challenging vasculopathy, which the latter often requires invasive histopathological diagnosis. In past decade, MR vessel wall imaging has quickly emerged as a non-invasive modality that greatly enhances radiologists’ capability of differentiating underlying aetiology. Not only it minimizes surgical trauma to patients, but also provide timely and accurate diagnosis for physicians to offer prompt appropriate treatment and avoid devastating outcomes. Tuen Mun Hospital, the neuroscience centre serving more than 1.3 million population in New Territories, Hong Kong, has been the pioneer in optimizing this novel modality within the district. In this article, we hope to share few interesting cases in our centre on how we utilize its advantage in solving some challenging cases. We would also discuss some common imaging pitfalls and tips on interpretation.

Cerebrovascular accidents remain one of the commonest causes of deaths in Hong Kong.^1^^,^^2^ While atherosclerotic disease is most common in the elderly group, the aetiology is much more diverse in younger patients, encompassing cardioembolic, primary or secondary vasculitic, or idiopathic causes.^3^ The differentiation of these causes is pivotal but challenging, and often requires extensive and invasive investigations. As of this moment, luminal imaging modalities such as CT/MR angiography remain the first-line non-invasive investigation. However, the *primum movens* of these diseases, that is, (non)infective and inflammatory changes which result in wall thickening and enhancement, are not well demonstrated. They could be of limited value and offered little insights for clinical teams in challenging cases.

Thanks to technological advancement, MR vessel wall imaging (MR-VWI) gradually gains its momentum worldwide in diagnosis of challenging vasculopathy cases. Unlike traditional luminal imaging, its main interest is more on wall morphology and characteristics which requires excellent imaging details. It differs with conventional MR sequences in multidimensional aspects, including but not only, magnetic field strength, isometric voxel size, field-of-view and turbo-spin-echo (TSE) factor for best spatial and contrast resolution.^4^^,^^5^ The imaging parameters in our neuroscience centre since 2022 were summarized in Table 1 and we would like to share a few cases where this novel imaging modality would be invaluable to radiologists.

**Table 1.** Parameters in our centre, mostly in-line with globally accepted parameters.

	MR-VWI
Sequences	T1-weighted, T2-weighted Diffusion-weighted imaging with apparent diffusion coefficient (DWI/ADC map) Susceptibility-weighted imaging (SWI) T1-weighted time-of-flight angiogram (T1-TOF) 3D-T1-weighted sequence with contrast 3D contrast-enhanced neck and cerebral angiogram
Magnetic field strength	3 Tesla (T)
Head coil channel	32
FOV (mm^3^)	200 × 250 × 171
Voxel size (mm^3^)	0.7 × 0.7 × 0.7
Turbo-spin-echo (TSE) factor	55
Contrast agent	Dotarem (0.1 mmol/kg)
Acquisition time	8 min 5 s
Black blood post-processing	VISTA

These measures are to achieve as good as possible spatial, contrast resolution, and signal-to-noise (SNR) ratio.

## Case 1

A 30-year-old lady with relatively good past health suffered from recurrent episodes of unilateral left upper and lower limb weakness and numbness, which are self-resolving within each episode. She has no loss of consciousness or focal neurological symptoms. Her Glasgow Coma Scale was 15/15 and physical examinations show no unilateral neurological signs. Her admission CT of the brain shows no abnormalities. Initial blood tests were unremarkable, and electroencephalogram (EEG) shows episodic right fronto-temporal sharpish slow wave.

**Figure 1.**
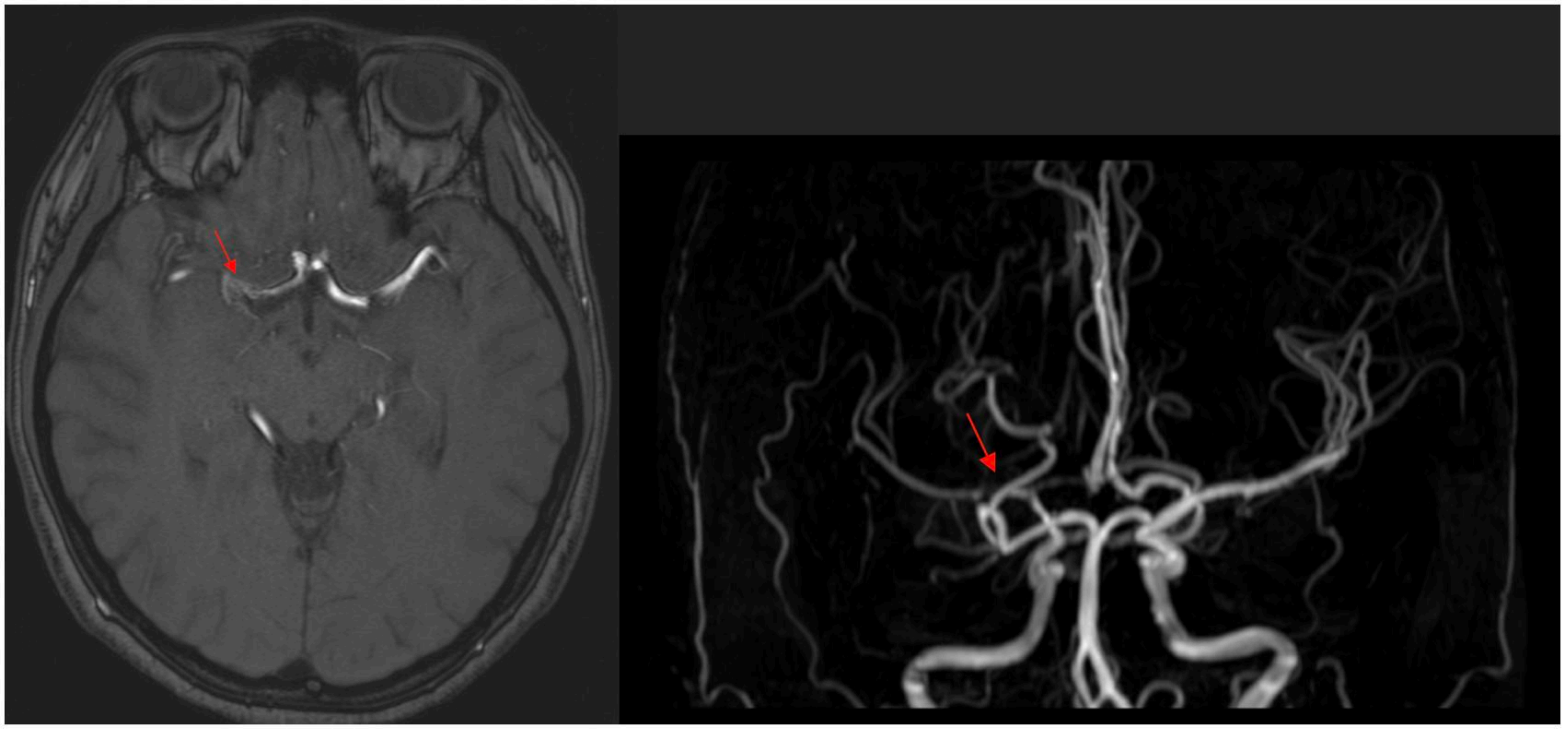
T1-weighted and 3D time-of-flight (TOF) show severe stenosis of M1, M2 segment of right middle cerebral artery (red arrow). No narrowing noted in contralateral side.

**Figure 2.**
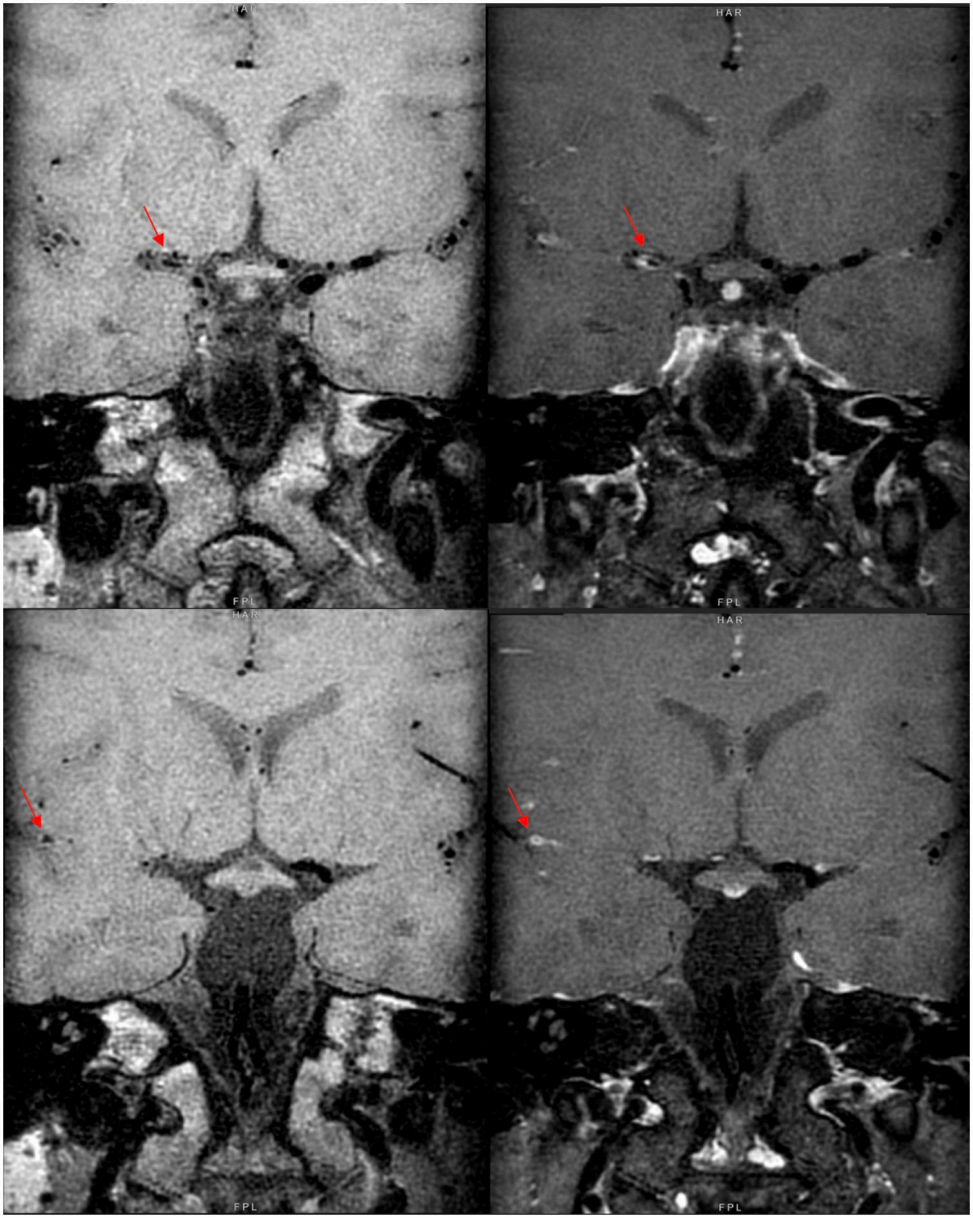
Pre-contrast (left) and post-contrast (right) MR-VWI — concentric luminal wall thickening and enhancement in M1 to proximal M2 segments of right middle cerebral artery (red arrow).

She had multiple admissions in view of recurrent minor-stroke like symptoms and further MR angiogram demonstrated multifocal, segmental bead-like attenuation and stenosis in M1 segment of right middle cerebral artery (MCA) as in Figure 1. VWI offers extra radiological features of luminal narrowing due to wall thickening in these segments shows concentric wall enhancement, which are compatible with vasculitis as in Figure 2.

Her follow-up blood tests show atypical antineutrophil cytoplasmic antibodies (ANCA) and negative findings in rest of autoimmune panels and lumbar puncture workup. She was treated as intracranial vasculitis and prescribed with high dose immunosuppressant (Prednisolone, Cyclophosphamide and Rituximab) with gradual tapering down. Follow-up MR-VWI shows minimal improvement in right MCA luminal attenuation and clinically she is symptom-free.

## Case 2

A 14-year-old boy previously suffered from Burkitt lymphoma with primary gastrointestinal (GI) tract tumour, which was excised in 2017 with complete remission in the same year, presented to Accident and Emergency Department with sudden headache, drowsiness and vomiting. His Glasgow Coma Scale was 11/15 and showed no focal neurological signs. Basic blood panels revealed elevated white cell count (15.5, normal 3.9-10.7) but otherwise unremarkable.

**Figure 3.**
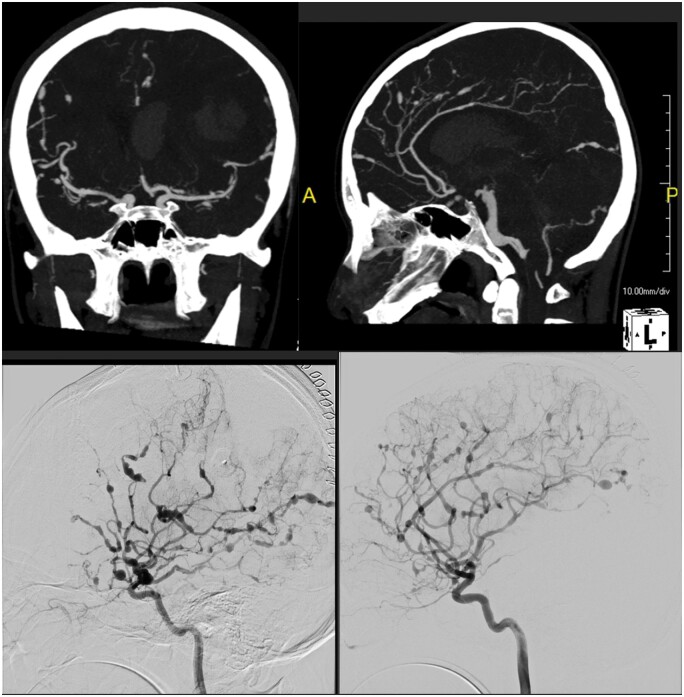
CT angiogram and digital subtraction angiogram show severe bead-like luminal attenuation in bilateral anterior and posterior circulations, predominantly in medium and small sized arteries.

On presentation his CT of brain revealed large left parietal haemorrhage with intraventricular extension and cerebral oedema. Subsequent CT angiogram and digital subtraction angiogram confirm extensive fusiform and saccular aneurysmal dilation, predominantly in medium and small sized arteries over bilateral anterior and posterior circulation in Figure 3. Fusiform dilation of basilar artery was also noted.

**Figure 4.**
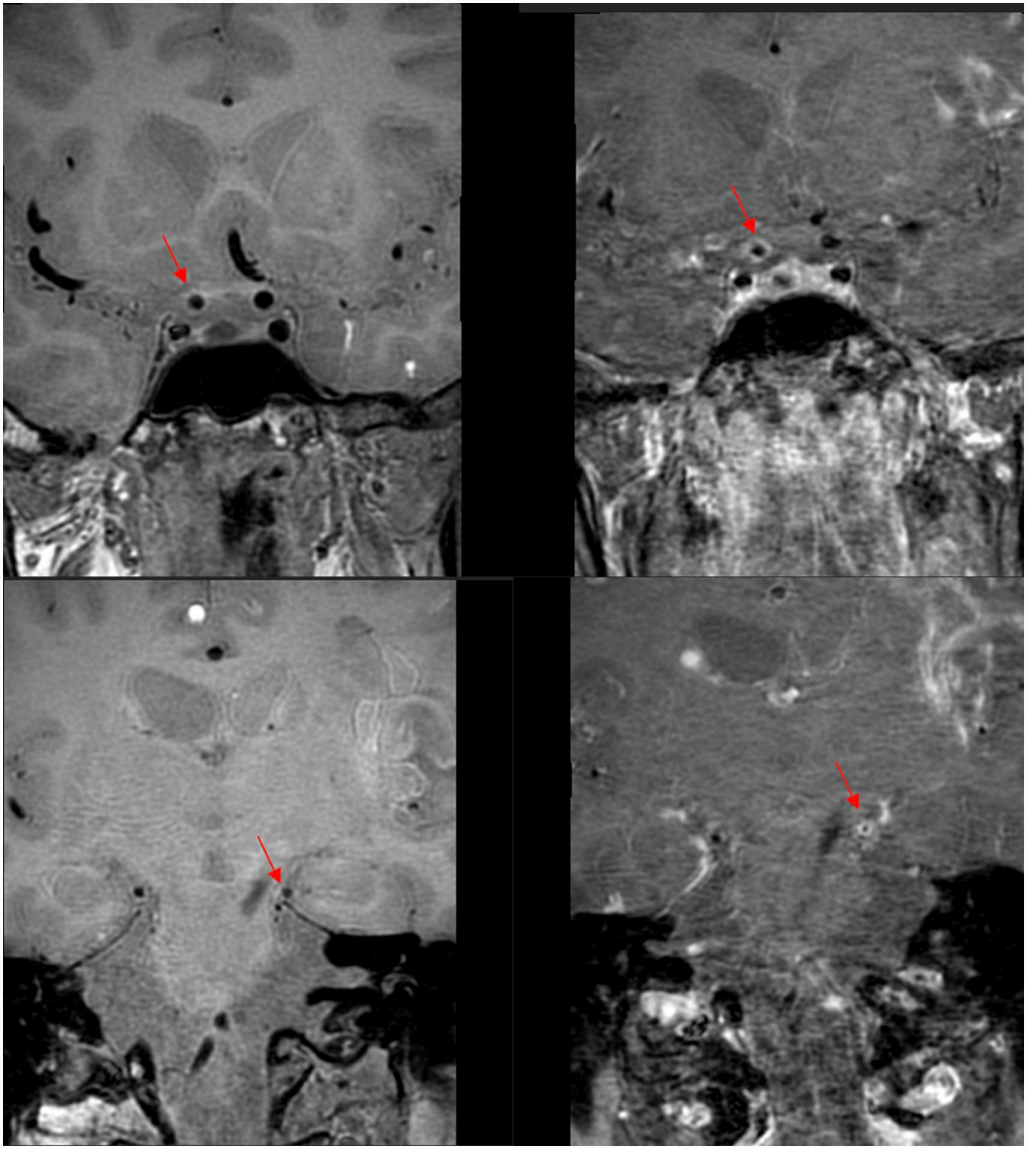
Paired pre-contrast (left) and post-contrast (right) MR-VWI show concentric luminal wall thickening and enhancement in major cerebral arteries, consistent with features of vasculitis (red arrows).

MR-VWI revealed concentric vessel wall thickening and enhancement in bilateral internal, middle and posterior cerebral arteries, consistent with intracranial vasculitis in Figure 4. Cerebrospinal fluid culture on the same day showed positive human herpes virus 6 growth (HHV6) and extensive 18 fluorodeoxyglucose (18FDG)-positron emission tomography of whole body showed no FDG-avid lesions in the rest of the body. Subsequent genetic study confirmed the diagnosis of X-linked lymphoproliferative disease. This is an entity of extremely rare incidence, with approximately one to three in a million boys and its relation to central nervous system (CNS) vasculopathy were only covered in few case reports globally.^6–8^ It is believed that pathogenesis was related to autoimmune response to viral infection (commonly human herpes virus/Epstein-Barr virus/human immunodeficiency virus). Despite intravenous immunoglobulin and immunosuppressants, he had little neurological improvement with recurrent episodes of thoracic and intraabdominal sepsis, and he eventually succumbed 3 months later.

## Case 3

**Figure 5.**
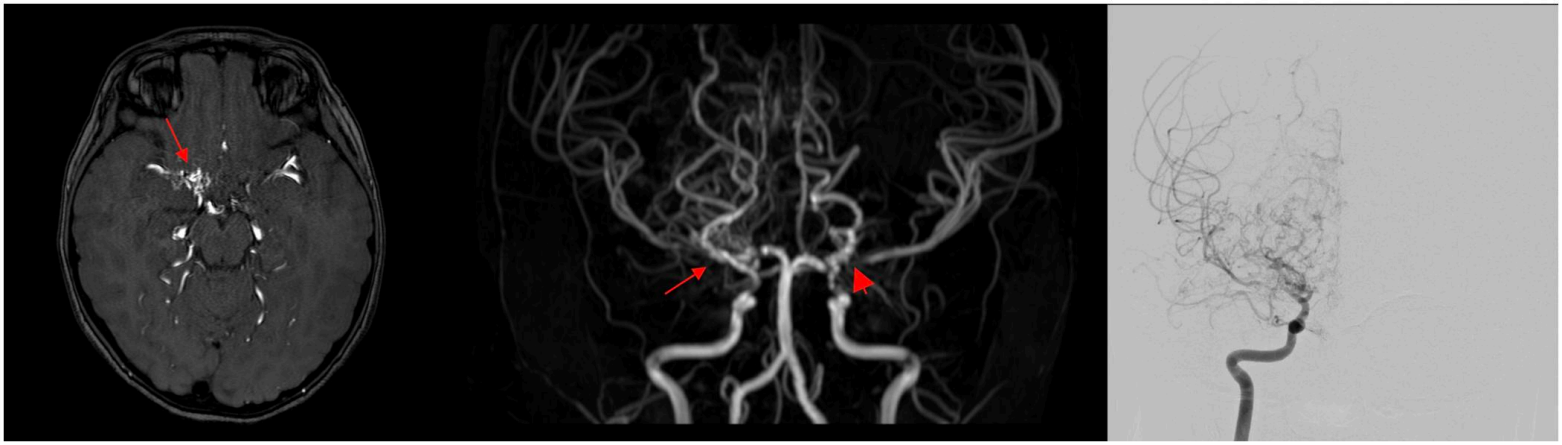
T1-weighted and 3D time-of-flight (TOF) MR angiogram—severe stenosis in M1 segment of right middle cerebral artery, and puff-of-smoke appearance in right lenticulostriate arteries (red arrow). Left M1 segment stenosis is less involved (arrowhead). Digital subtraction angiogram of right internal carotid artery shows severely attenuated M1 segment with opening of collaterals in right lenticulostriate perforators, compatible with Moyamoya phenomenon.

**Figure 6.**
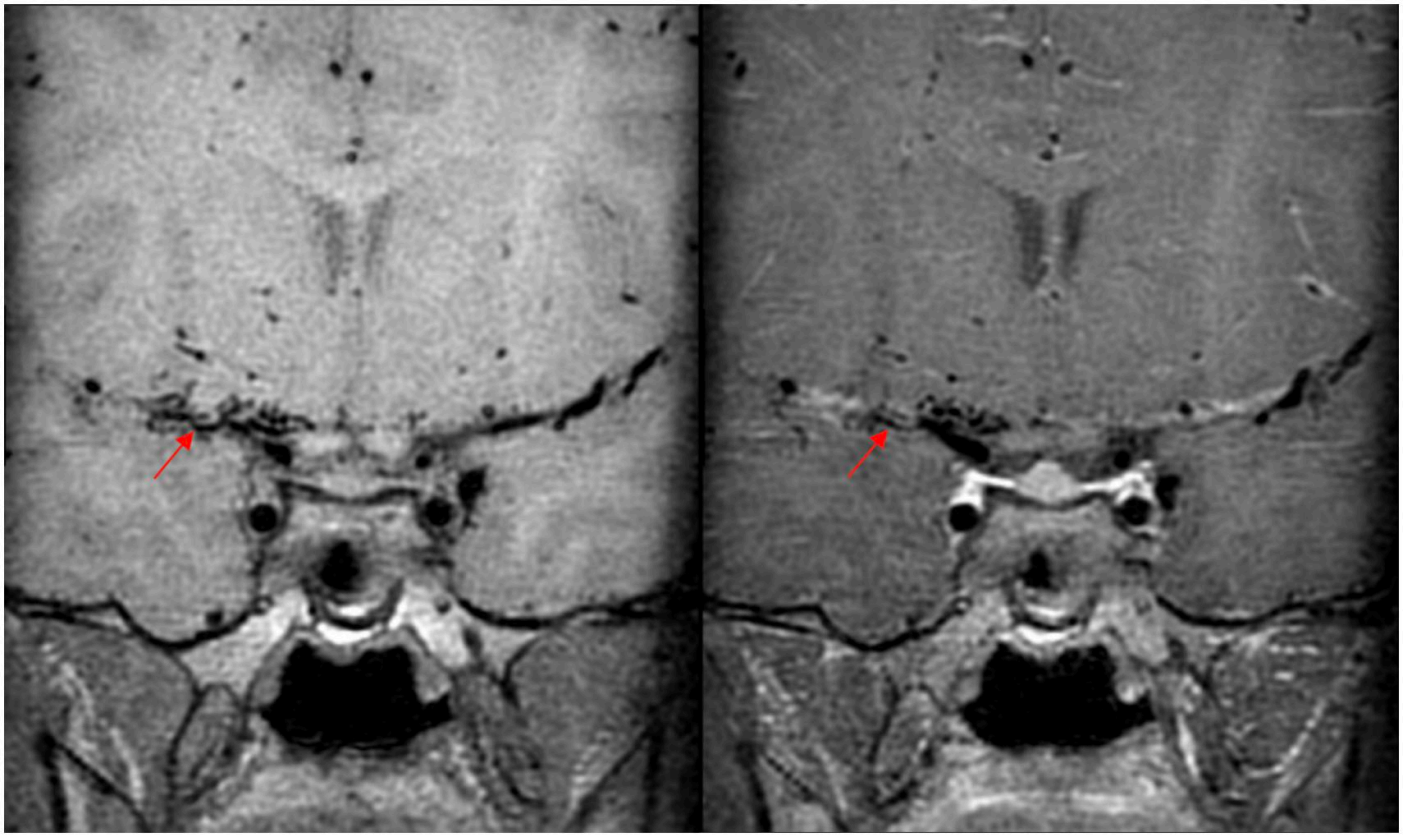
Pre-contrast (left) and post-contrast (right) MR-VWI—concentric luminal wall thickening without enhancement (red arrows); compatible with non-inflammatory vasculopathy as Moyamoya phenomenon.

A 9-year-old girl with multiple episodes of left hemiparesis and numbness since 2021, presented to neurology clinic for follow-up and revealed increasing episode and frequency of limb twitching. No focal neurological signs and her Glasgow Coma Scale was 15/15. Physical examination, blood tests and initial CT of the brain were unremarkable. MR-VWI performed in view of young onset stroke-like symptoms and revealed bilateral near-total occlusion of anterior cerebral arteries, and severely attenuated middle cerebral arteries with abundant collaterals opening as in Figure 5. No wall thickening nor enhancement in the affected segments as in Figure 6, which are most consistent with Moyamoya phenomenon.^9^ Digital subtraction angiogram confirmed above MR findings and patient underwent bilateral external carotid-internal carotid (EC-IC) bypass with lifelong antiplatelet regime. She enjoyed full neurological recovery and is lost to follow-up due to immigration.

## Discussions

These cases shed light on the extra yet critical imaging clues that conventional luminal cross section images or angiography failed to offer. Luminal remodelling and wall enhancement pattern were described in multiple large-scale VWI articles worldwide.^4^^,^^5^ For instance, inflammatory vasculitis involves the lymphocytic infiltration of vessel walls, which typically demonstrates concentric wall thickening and enhancement. On the other hand, Moyamoya phenomenon refers to a chronic occlusive non-inflammatory, idiopathic vasculopathy, where no wall enhancement apart from luminal remodelling would be expected.^10^ Intracranial atherosclerotic disease (ICAD), another common disease entity relatively affecting the elderly (not in shown in this case series), would lack wall enhancement on top of multifocal eccentric negative luminal remodelling.

Apart from major application on wall morphology, there has been more ongoing research on extracranial vasculopathy. The major interest would fall into atherosclerotic plaque characterization and extracranial VWI. Currently, some propose the plaque enhancement pattern and degree of luminal narrowing would correlate with plaque rupture risk, hence estimate the risk of cerebrovascular accident despite insufficient large scale randomized-control trials up to this moment.^11^ Another trend of MR-VWI focuses on characterization of extracranial vasculopathy, particularly for giant cell arteritis (GCA) patients. Some studies quoted optimized MR-VWI protocols offer excellent localization for neurology physicians or neurosurgeons to enhance diagnostic sensitivity of vessel biopsy with targeted sampling,^12^ which is out of the scope of this case series.

**Figure 7.**
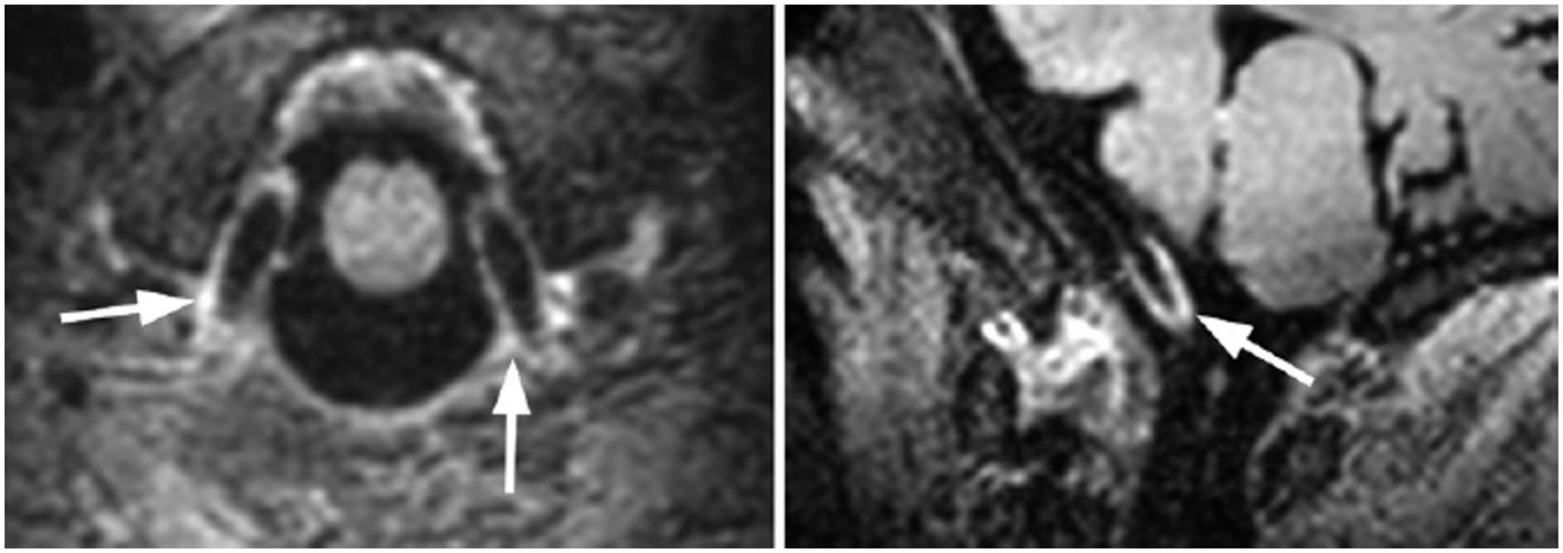
MR-VWI of a 43-year-old healthy individual demonstrating concentric wall enhancement in V4 segment of vertebral artery due to vasa vasorum.

All imaging tools are not without limitations, and MR-VWI is no exception. There are three commonly recognized pitfalls which could affect radiologists’ interpretations. The first would be vasa vasorum. These are venous plexus normally found in extracranial segments and migration to intracranial segments would be prominent by ageing in Figure 7. Extracranial arteries are therefore normally not within VWI interest and explains its relatively small field-of-view (FOV) size, targeting intracranial arteries within circle of Willis.

**Figure 8.**
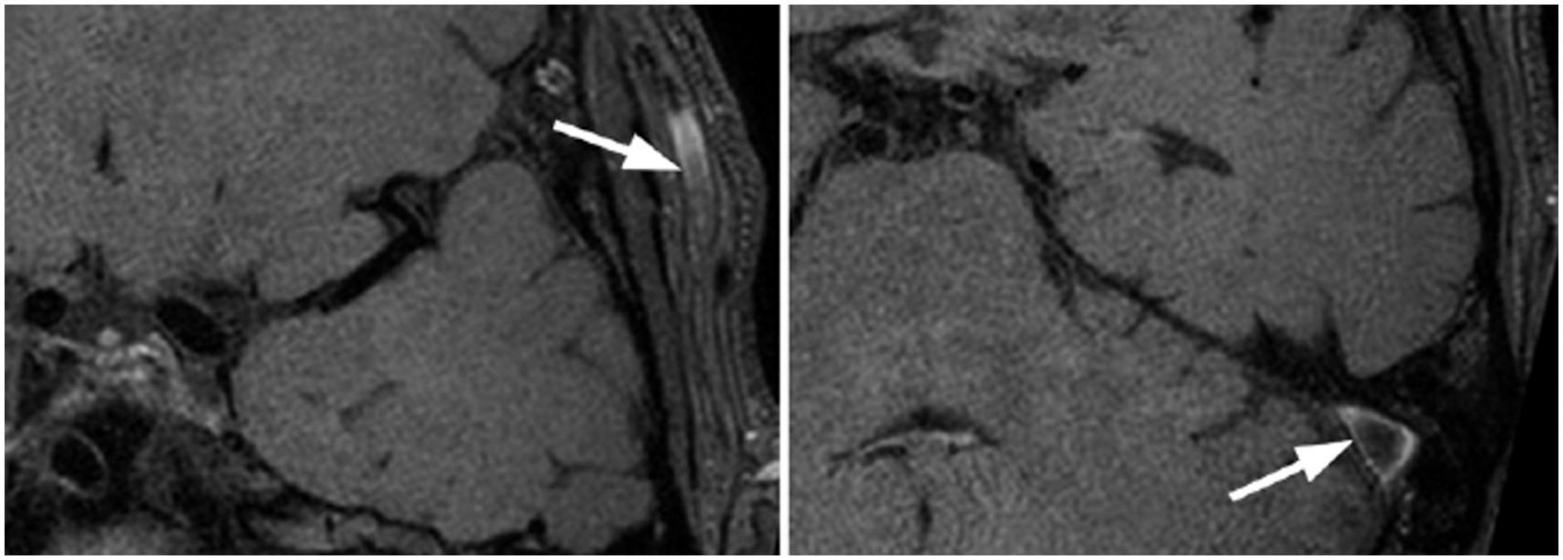
MR-VWI of a 38-year-old healthy individual, with apparent wall enhancement in extracranial veins and sigmoid sinuses.

**Figure 9.**
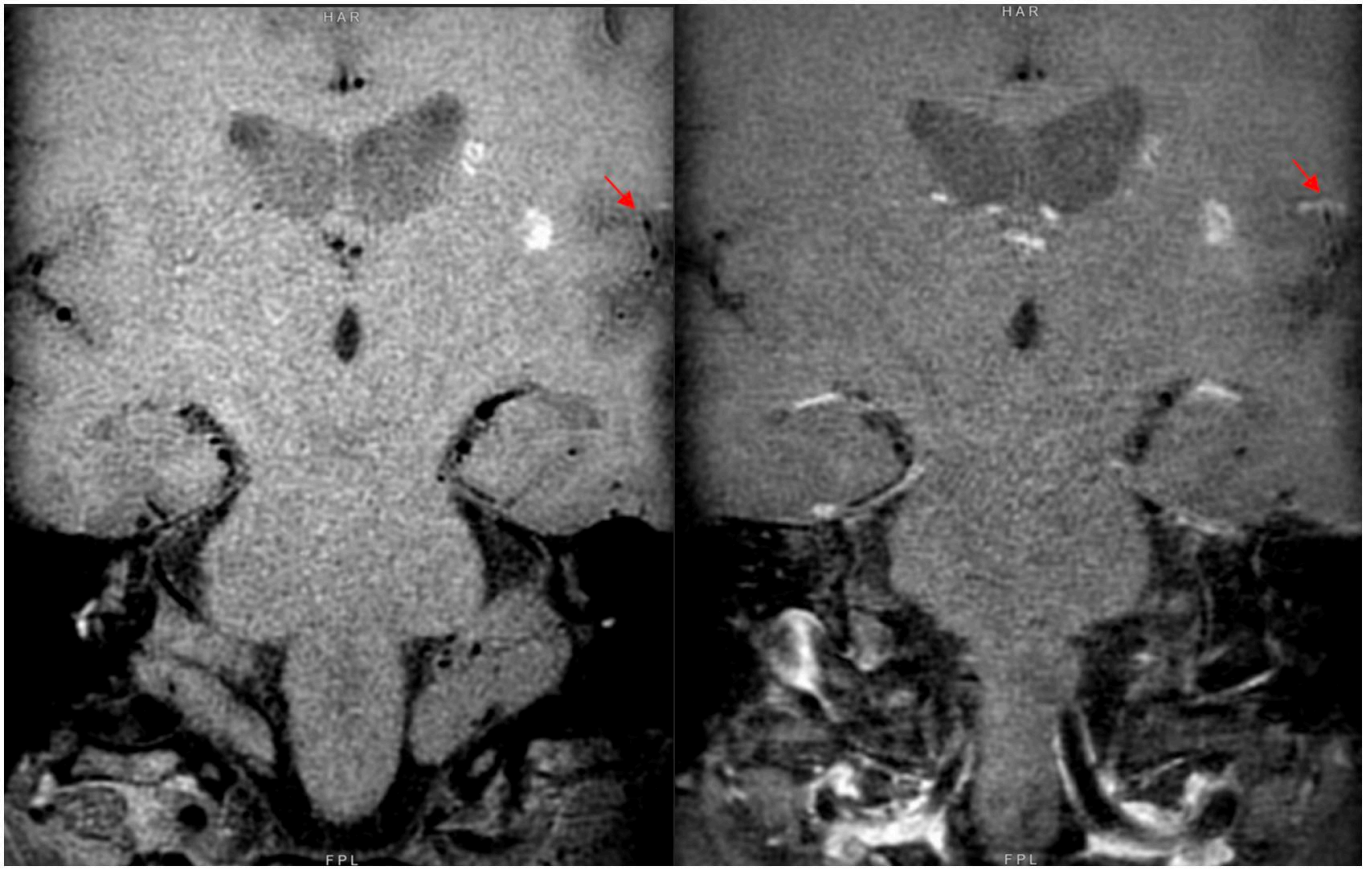
MR-VWI of a 57-year-old normal patient. Notice the apparent wall enhancement in distal M3 segment of left middle cerebral artery shows no luminal narrowing, likely due to increased susceptibility to slow flow artefact in distal arterial flow disruption (red arrows).

The second one would be artefacts by enhancing deep cerebral veins. The reduced venous luminal flow compared with arterial flow results in incomplete signal void on pre-contrast study, due to fast black blood void technique, which in turn results in venous contamination and falsely taken as wall enhancement in inexperienced hands, as in Figure 8. Similar slow flow artefact would be significant in distal arterial branches as well due to their turbulent flow on VWI in contrast to laminar flow in luminal centre, resulting in incomplete black blood voiding in Figure 9. We recommend correlation with original T1-time-of-flight (TOF) images and correlate with arterial flow frame-by-frame to reduce overcall.

**Figure 10.**
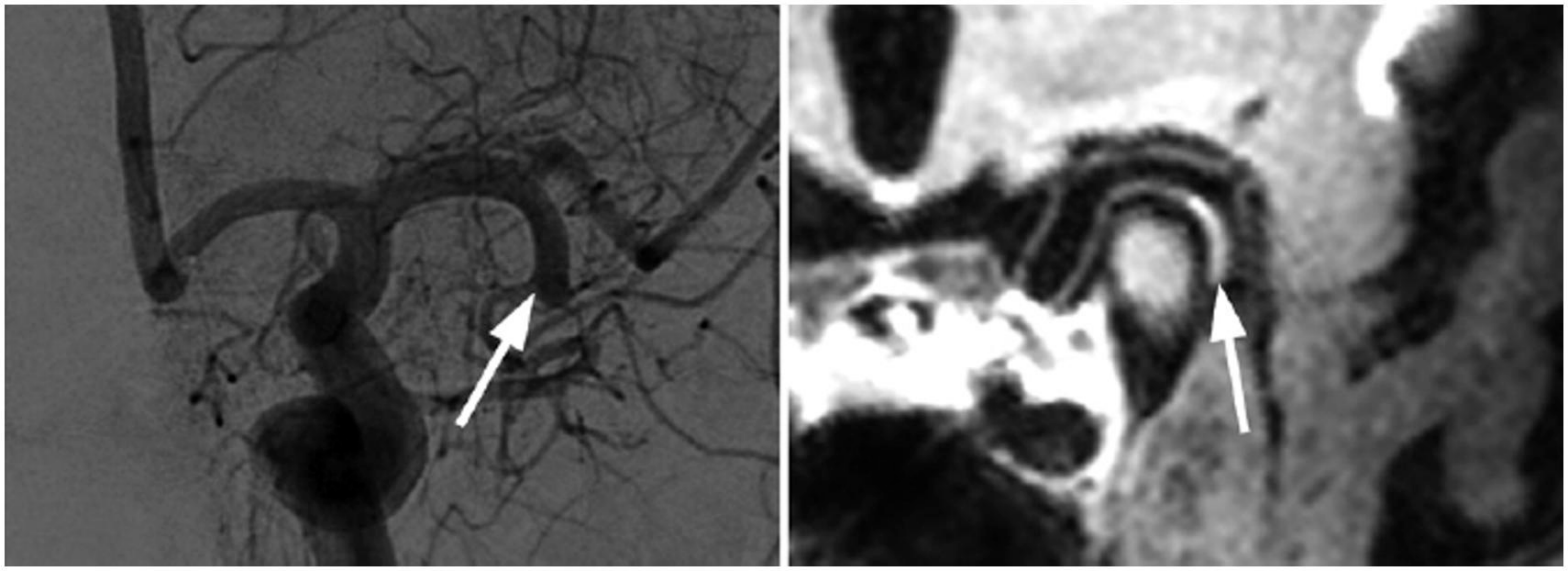
Left: acute left M1-2 thromboembolic occlusion with subsequent intraarterial thrombectomy performed (not shown). Right: MR-VWI at day 22 post-procedure revealed eccentric wall enhancement after contrast administration at the same location as the thrombectomy site.

Finally, iatrogenic causes related to thrombus-induced fibrosis due to recent recanalization would cause significant false-positive. Many studies in the world have arrived the post-procedural wall enhancement, resembling with pattern normally depicted in acute vasculitis in Figure 10.^13^ Exact mechanism remains a mystery but is believed the fibrin-rich thrombus stimulate collaterals walls rich with reactive changes.

With all the interpretation challenges, one would expect potential overcall when only few imaging features were used as diagnostic criteria. Hence, we propose extreme meticulous judgement and correlation with all MR sequences should radiologists has suspicion, which include careful examination with plain sequences, luminal imaging, and last but not least, clinical presentation. Wall reactive changes should only be regarded as genuine based on negative luminal attenuation and wall thickening.

## Conclusion

Timely and precise diagnosis of vasculopathy in young patients presenting with recurrent acute stroke-like symptoms is paramount, which greatly determine their neurological outcome and as such, their future quality of life. MR-VWI has undoubtedly proven its capability as a powerful non-invasive diagnostic modality that radiologists should leverage its maximum potential. Although vessel biopsy remains the gold standard for histological diagnosis, VWI serves as a non-invasive but strong evidence of vessel wall pathologies, as well guides sampling of diseased vessels during biopsy. Despite its potential interpretation pitfall and limitations, we would continue optimize its diagnostic accuracy and explore its other potential use with careful examination.

## Learning points

- MR-VWI emerges as a novel, highly sensitive, non-invasive diagnostic modality for intracranial vasculopathy with excellent spatial and contrast resolution. More research are currently underway with higher magnetic field scanners and scanning protocol to optimize image quality and minimize artefacts. Some centres proposed extending its application to atherosclerotic plaques characterization and extracranial arterial assessment for guide potential surgical site for histopathological diagnosis in cases of large to medium-sized vasculitis, such as giant cell arteritis (GCA).
- We recommend careful selection of cases who undergo MR-VWI as it requires experienced hands and thorough understanding. In our centre practice, it is mainly reserved for young patients with recurrent or progressive ischaemic stroke.
- Careful evaluation of vessel wall condition is paramount before committing genuine vasculopathy. It is not uncommon to encounter artefacts in well-designed scanning protocols as above mentioned. Whenever encountering suspected luminal narrowing, make sure to correlate with concomitant luminal imaging and clinical presentation. Comments on wall thickening pattern with plain study should always precedes enhancement pattern to minimize common artefacts.
- Radiologists should be cautious of its common pitfall and correlate with clinical information when in doubts to avoid overcall genuine pathology.

## Data Availability

Data sharing not applicable as no datasets were generated.
